# Presurgical assessment of bariatric patients with the Patient Health Questionnaire (PHQ)—A screening of the prevalence of psychosocial comorbidity

**DOI:** 10.1186/s12955-015-0278-5

**Published:** 2015-06-10

**Authors:** Patrick H. Alizai, Maren K. Akkerman, Daniel Kaemmer, Florian Ulmer, Christian D. Klink, Sabine Ernst, Klaus Mathiak, Ulf P. Neumann, Volker Perlitz

**Affiliations:** Department of General-, Visceral- and Transplantation Surgery, RWTH Aachen University Hospital, Pauwelsstr. 30, 52074 Aachen, Germany; Department of Medicine, Luisenhospital Aachen, Academic teaching hospital of the RWTH Aachen University, Boxgraben 99, 52064 Aachen, Germany; Department of Surgery, St. Elisabeth Hospital Geilenkirchen, Martin-Heyden-Str. 32, 52511 Geilenkirchen, Germany; Institute of Medical Statistics, RWTH Aachen University Hospital, Pauwelsstr. 30, 52074 Aachen, Germany; Department of Psychiatry, Psychotherapy and Psychosomatics, RWTH Aachen University Hospital, Pauwelsstr. 30, 52074 Aachen, Germany

**Keywords:** Obesity, Bariatric surgery, Patient health questionnaire, Computerized, Mental health disorder, Self reporting

## Abstract

**Background:**

Bariatric surgery has gained increasing relevance due to the dramatic rise in morbid obesity prevalence. A sound body of scientific literature demonstrates positive long-term outcome of bariatric surgery in decreasing mental and physical health morbidity. Still, there is a need for a manageable presurgical screening to assess major mental disorders. The aim of this study was to assess the frequency of common psychiatric syndromes in bariatric surgery candidates using a computerized version of the Patient Health Questionnaire (PHQ).

**Methods:**

In a prospective cohort study from August 2009 to July 2011 morbidly obese individuals seeking bariatric treatment were evaluated for mental health disorders using the PHQ (computerized German version).

**Results:**

A total of 159 patients were included in this study. The median age of participants was 42 years, the median BMI was 49 kg/m^2^. The PHQ revealed a prevalence of 84 % for mental health disorders, 50 % of the participants had three or more mental health disorders. A high somatic symptom burden (46 %), depressive syndromes (62 %) and anxiety disorders (29 %) were the most frequent psychiatric syndromes. The median number of psychiatric syndromes was 3 for women and 1 for men (*p* = 0.007). No correlation between BMI and a single syndrome or the sum of syndromes was observed.

**Conclusion:**

84 % of the patients seeking bariatric treatment were screened positive for at least one mental health disorder. The computerized PHQ with automated reporting appears to be a useful instrument for presurgical assessment of bariatric patients in routine medical settings.

## Background

The prevalence of overweight and obesity is increasing globally in both developed and developing countries [1]. Obesity is associated with type 2 diabetes, hypertension, non-alcoholic steatohepatitis, sleep apnea syndrome, and various other co-morbidities, which may lead to further morbidity and mortality [2]. Specific sequels associated with treatment and management of morbid obesity account for 5–10 % of healthcare spending in the US [1, 3]. Obesity ranks second to smoking in avoidable cases of death, decreasing life expectancy by an estimated 20 years [4]. There is widespread consensus among health care professionals that obesity can be attributed to physiological, psychological, and social factors [5]. However, even in the presence of distinct mental health disorders which may lead to or augment obesity, conservative therapies of mental health disorders fail to correct morbid obesity in the long run. So far, only bariatric surgical methods have been demonstrated to result in long-term excess weight losses of 40 to 70 % [6, 7]. Moreover, bariatric surgery yields improvement in obesity-related co-morbidities and improves post-surgical quality of life [6, 8, 9].

Several variables affect the amount of excess body weight loss and mechanisms responsible for the effects of surgery are not entirely understood [10]. Factors that may be negatively associated with postoperative weight loss include preoperative BMI, super-obesity, and mental disorders [11]. Mental health co-morbidity has repeatedly been shown to impact postsurgical outcome [12, 13]. The identification of psychopathological predisposing factors possibly responsible for poorer treatment outcomes suggests employing a reliable mental health screening in bariatric surgery. Since the diagnosis of frequent mental health conditions such as depression, anxiety, eating or somatoform disorders is difficult for surgeons compared to mental health care professionals, there is ample need for assistance with these diagnoses to avoid underdiagnosis [14, 15]. Various psychological test procedures are commonly used in the presurgical evaluation process; however, no uniform guidelines exist for the psychological assessment of surgery candidates [16].

The Patient Health Questionnaire (PHQ) is a standardized self-report instrument which was designed to screen for the most frequent mental health disorders in primary health care [17] (German version [18–20]). The aim of this study was to assess the frequency of common psychiatric syndromes in patients seeking bariatric surgery using a computerized version of the Patient Health Questionnaire (PHQ).

## Patients and methods

This study was conducted at the interdisciplinary bariatric centre of the RWTH Aachen University Hospital between August 2009 and July 2011. Participants were bariatric surgery candidates with body mass indices of > 40 kg/m^2^ or > 35 kg/m^2^ and weight-related co-morbidities. Exclusion criteria were minority, absence of written consent and illiteracy. Bariatric surgery candidates (292 individuals) firstly attended an interdisciplinary information meeting in order to obtain comprehensive information on the medical backgrounds of morbid obesity and its bariatric treatment. Following each meeting, attendants were given the opportunity of computerized mental health screening. One hundred eighty-three individuals decided to participate in this mental health screening. The screening was performed employing a software package collecting sociodemographic data on the patient’s history (Procalysis^(R)^ eTest Anamnesis, Simplana GmbH, Aachen, Germany) and digital PHQ testing. All data were saved in an internal database and secured using 128 bit encryption technology.

This study was approved by the local RWTH Aachen University Ethics Committee and written informed consent was obtained from all patients.

### PHQ

The Patient Health Questionnaire (German version) is a standardized questionnaire which was designed to screen for the most frequent mental health disorders in primary health care. To meet our purposes, we employed the full version of the PHQ which contains 78 items allowing screening of the following mental health disorders: major and other depressive syndromes (9 items), panic and other anxiety syndromes (15, resp. 7 items), somatic symptom burden (13 items), bulimia nervosa and binge eating disorder (8 items), alcohol abuse and alcohol dependency (6 items), and psychosocial functioning and stressors (10, resp. 10 items). Some of the modules contain filter items which may shorten the test. Skipping of a displayed question was not possible in the computerized version. The requested time frame depends on the module and varies between 2 weeks (depressive symptoms) and 6 months (alcohol consumption). The PHQ has been extensively validated in various patient populations [17–20]. The internal consistency for continuous scales is for the depression module r = 0.88 and r = 0.79 for the somatization module. Test-retest-reliability of the depression module ranges between ICC = 0.81 and ICC = 0.96. The depression scale of the PHQ was shown to be sensitive to change processes which allows its use in longitudinal studies assessing efficacy of therapy effects [18, 21]. The PHQ test was applied in a computer-based version. Results are instantly computed by the software and are available to therapists and patients upon completion of data entry.

### Statistical analysis

Statistical evaluation was carried out using the SAS 9.2 software (SAS Institute, Cary, NC, USA). Values are presented as means and standard deviations or median and interquartile range if not otherwise specified. Significance was calculated using the Chi-square test, Wilcoxon signed-rank test, Fisher’s Exact test and t-test. A *p* < 0.05 was considered significant.

## Results

From a total of 292 individuals who had attended the information meeting about bariatric surgery, 183 (62.7 %) decided to participate in the digital mental health screening. One hundred fifty-nine candidates (54.5 %) completed the informed written consent permitting evaluation of their digital psychometric and sociodemographic data in this study. Completion of the electronic version of the PHQ test took on average 8.5 ± 3.2 min (range 4–20.5 min). Due to filter items, 60 items (range 47–78) were answered on average.

The participants were aged 42 ± 11 years (range 19–68 years), 65.4 % of which were females (Table 1). The mean BMI of the participants was 49 ± 8 kg/m^2^. Sociodemographic data showed for the educational background that 3.1 % of candidates had not completed secondary education, 61.0 % were secondary school graduates, 25.2 % had completed high school, and 10.7 % were university graduates. As for the family status, 50.3 % of candidates were married, 17.6 % lived in a partnership, 8.8 % were divorced or widowed, and 22.6 % were single. The vocational status showed that 39.0 % of candidates were unemployed, 14.5 % were early retired, 8.2 % were workers, 34.0 % were employees, and 4.4 % were self-employed (Table 1).

**Table 1:** Sociodemographic data

Participants	All (*n* = 159)	Women (*n* = 104)	Men (*n* = 55)
Age (years)	42.2 (±10.7)	41.2 (±11.1)	44.0 (±9.6)
BMI (kg/m^2^)	49.3 (±8.4)	49.1 (±8.5)	49.8 (±8.4)
Education			
No graduation	5 (3.1 %)	4 (3.9 %)	1 (1.8 %)
Secondary school	97 (61.0 %)	60 (57.7 %)	37 (67.3 %)
High school	40 (25.2 %)	31 (29.8 %)	9 (16.4 %)
University	17 (10.7 %)	9 (8.7 %)	8 (14.6 %)
Marital Status			
Single	36 (22.6 %)	25 (24.0 %)	11 (20.0 %)
Partnership	28 (17.6 %)	18 (17.3 %)	10 (18.2 %)
Married	80 (50.3 %)	52 (50.0 %)	28 (50.9 %)
Divorced	14 (8.8 %)	8 (7.7 %)	6 (10.9 %)
Widowed	1 (0.6 %)	1 (1.0 %)	0 (0 %)
Employment			
Unemployed	62 (39.0 %)	38 (36.5 %)	24 (43.6 %)
Worker	13 (8.2 %)	6 (5.8 %)	7 (12.7 %)
Employee	54 (34.0 %)	38 (36.5 %)	16 (29.1 %)
Self-employed	7 (4.4 %)	6 (5.8 %)	1 (1.8 %)
Early Retired	23 (14.5 %)	16 (15.4 %)	7 (12.7 %)

**Fig. 1:**
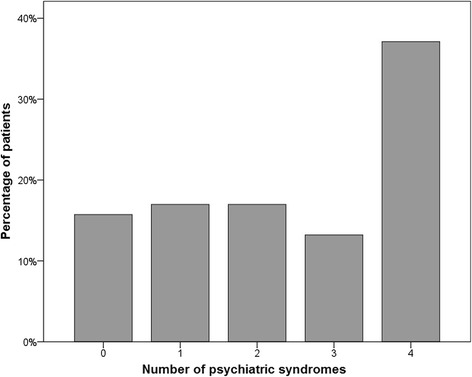
Distribution of psychiatric syndromes: no disorder (15.7 %), a single disorder (17.0 %), two (17.0 %), three (13.2 %), four or more than four psychiatric syndromes (37.1 %). About half of the patients (50.3 %) applying for bariatric surgery exhibited mental health problems in at least three areas

Using the PHQ, the following distribution of psychiatric syndromes was observed: 25 candidates (15.7 %) showed no disorder, 27 candidates (17.0 %) were screened positive for a single syndrome, 17.0 % for two syndromes, and 50.3 % for three or more (Fig. 1). 84.3 % of the participants were screened positive for at least one mental health disorder. The mental health status exhibited in 58.5 % a high somatic symptom burden, panic disorders in 18.2 %, major depressive syndromes in 26.4 %, other depressive disorders in 44.7 %, bulimia nervosa in 10.7 %, binge eating syndrome in 20.8 %, anxiety disorders in 45.3 %, and alcohol syndrome in 6.3 % (Table 2). Median number of “PHQ diagnoses” was gender dependent and significantly different: 3 for women and 1 for men (*p* = 0.007). A high somatic symptom burden and anxiety disorders were detected significantly more often in women (*p* = 0.015, and *p* = 0.003, resp.). No correlation between BMI and a single syndrome or the sum of syndromes was observed. The distribution of syndromes and employment status was analysed and results were displayed in Table 3. Unemployed participants (39.0 %) and early retirees (14.5 %) were subsumed as ‘unemployed’. Somatic symptom burden (*p* = 0.019), other depressive disorder (*p* = 0.024) and panic disorder (*p* = 0.024) were significantly more often in the unemployed group.

**Table 2:** Gender-specific distribution of psychiatric syndromes

Syndrome	All	Women (*n* = 104)	Men (*n* = 55)	*p*-value
	(*n* = 159)			
Somatic symptom burden	93 (58.5 %)	68 (65.4 %)	25 (45.5 %)	0.015*
Major depressive syndrome	42 (26.4 %)	29 (27.9 %)	13 (23.6 %)	0.563*
Other depressive disorder	71 (44.7 %)	50 (48.1 %)	21 (38.2 %)	0.233*
Panic disorder	29 (18.2 %)	21 (20.2 %)	8 (14.6 %)	0.381*
Anxiety disorder	72 (45.3 %)	56 (53.9 %)	16 (29.1 %)	0.003*
Bulimia nervosa	17 (10.7 %)	13 (12.5 %)	4 (7.3 %)	0.310*
Binge eating disorder	33 (20.8 %)	25 (24.0 %)	8 (14.6 %)	0.160*
Alcohol syndrome	10 (6.3 %)	4 (3.9 %)	6 (10.9 %)	0.081*
Median number	2 (IQR = 3)	3 (IQR = 3)	1 (IQR = 2)	0.007^a^

^a^ Wilcoxon signed-rank test

* Chi-square test

**Table 3:** Employment status and distribution of psychiatric syndromes

Syndrome	All	Employed	Unemployed	*p*-value
	(n = 159)	(n = 74)	(n = 85)	
Somatic symptom burden	93 (58.5 %)	36 (48.6 %)	57 (67.1 %)	0.0188*
Major depressive syndrome	42 (26.4 %)	15 (20.3 %)	27 (31.8 %)	0.1010*
Other depressive disorder	71 (44.7 %)	26 (35.1 %)	45 (52.9 %)	0.0243*
Panic disorder	29 (18.2 %)	8 (10.8 %)	21 (24.7 %)	0.0236*
Anxiety disorder	72 (45.3 %)	32 (43.2 %)	40 (47.1 %)	0.6297*
Bulimia nervosa	17 (10.7 %)	10 (13.5 %)	7 (8.2 %)	0.2827*
Binge eating disorder	33 (20.8 %)	16 (21.6 %)	17 (20.0 %)	0.8014*
Alcohol syndrome	10 (6.3 %)	5 (6.8 %)	5 (5.9 %)	1.000^b^
Median number	2 (IQR = 3)	2 (IQR = 2)	2 (IQR = 3)	0.0345^a^

Employed participants and early retirees were subsumed as ‘unemployed’

^a^ Wilcoxon signed-rank test

^b^ Fisher’s exact test

* Chi-square test

## Discussion

Bariatric surgery has been on the rise over the past two decades. Increasing prevalence of obesity and the development of laparoscopic surgery have contributed to this increase, but above all bariatric surgery has proved to be the most effective therapy for morbid obesity. Despite an overall improvement, long-term postsurgical weight loss is fraught by some setbacks as approximately 20 to 30 % of patients regain weight following surgery [16, 22]. To some portion this is due to the lack of changes in lifestyle and eating behaviours, which not only comprise surgical outcome by weight regain and malnutrition, but also the deterioration of pre-existing mental health disorders have being reported [16, 23]. Surgical outcome can be impaired both by physiological postoperative changes and psychosocial factors [12, 22, 24]. Therefore, presurgical psychological evaluation of bariatric surgery candidates is recommended by the guidelines and frequently demanded by insurance companies [1, 25, 26]. However, no standards of how best to assess bariatric surgery candidates have currently been defined [27, 28]. In this study, we present our results on screening for mental health disorders of morbidly obese individuals seeking bariatric treatment using the PHQ. Originally designed to screen for eight different mental health disorders in primary health care, the PHQ has been validated in numerous studies and is a well-accepted test instrument [17, 18, 21]. It allows assignment of patients to one of four subgroups reflecting the severity of mental health disorders. Assessing the severity of symptoms has been shown to impact stronger than the specificity of diagnoses [22].

Employing the PHQ in a computerized version, almost 85 % of the participants were screened positive for a least one psychiatric syndrome, which is much in keeping with data reported in existing literature for the lifetime history of major mental disorders in the morbidly obese [22, 29, 30]. More than 50 % of the tested candidates exhibited three or more mental health disorders, which underlines the need for a differentiated psychological evaluation. In particular, obese patients seeking bariatric surgery are known to have markedly higher prevalence rates of psychopathologies than normal weight individuals [16]. Owing to the bio-psycho-social model proposed by Engel [31], factors triggering morbid obesity may be associated to biological, psychological, or social factors. There is ample reason to assume the apparent physical symptom “obesity” to result from a hazardously intertwined interaction between these factors. The hazardous nature of this interaction is also evident since the correction of psychic or social problems mostly fails to curtail the exacerbation of obesity once weight has climbed beyond 40 kg/m^2^ [22]. The PHQ may qualify as a test tool since it not only screens for mental health disorders but also includes physical symptoms pertaining to mental health disorders such as pain. Nearly 60 % of the participants in this study exhibited a high somatic symptom burden, with pain being the most frequently presented complaint. It is likely that the high frequency of somatic complaints can be attributed to the physical consequences of morbid obesity, e.g. musculoskeletal pain or shortness of breath. Increased prevalence rates of somatoform disorders in overweight and obese individuals were also reported in various other studies [32, 33].

Anxiety disorders and depressive syndromes were the second most common syndromes found in our study population. A positive association between obesity and anxiety disorders was also described by some other authors [34, 35]. Obese individuals have a life-time prevalence of depression ranging from 29 to 51 % [36–38] and obesity is associated with a higher risk of possible depression [39]. In a population-based study of 40,000 US adults, the risk of major depression in persons with obesity grade III was nearly five times that in normal weight individuals [40]. Furthermore, patients who remain depressed following bariatric surgery, tend to report poorer postsurgical quality of life and weight loss [41]. Andersen and colleagues found that following bariatric surgery, anxiety and depression were reduced and that these changes were closely associated with improvements in self-reported physical health [42].

Numerous studies have focused on the eating behaviour of individuals seeking bariatric surgery and the most common and likely relevant condition is binge eating disorder [5]. Twenty percent of our study sample exhibited a binge eating disorder, which is in the range of prevalences reported for bariatric surgery candidates [38, 43]. Binge eating could have detrimental effects on surgical outcome as it is associated with greater weight regain [44, 45]. Therefore, some authors recommend binge eating as a marker of poorer outcome and an appropriate target for post-surgical intervention [45].

An important scale of the PHQ screens for alcohol syndrome which is a contraindication for bariatric procedures due to increased sensitivity to alcohol postoperatively. Furthermore, alcohol consumption may compromise weight loss following bariatric surgery [1, 46]. Kudsi and colleagues systematically assessed 653 patients seeking weight-loss surgery and reported alcohol abuse in 4 % of their candidates which corresponds to the results in our PHQ assessed patient cohort [46]. Other authors found alcohol abuse or alcohol dependency in more than 30 % of bariatric surgery candidates [38].

The male to female ratio in our cohort was 1:1.9, which is also a standard phenomenon in studies on bariatric surgery candidates [30, 47]. Males more seldom tend to request surgical treatment for their obesity, while females tend to more regularly seek effective methods for treating their disease [47, 48]. Further, the median number of PHQ syndromes was significantly higher in women than in men. In particular, a higher somatic symptom burden and anxiety disorders were found significantly more often in women than in men. These results are in line with other studies, which found more psychopathologies among women [47, 49]. Gender effects on motivation for bariatric surgery and psychological co-morbidity therefore need to be considered in presurgical evaluation of bariatric candidates.

It is striking that 39 % of the participants were unemployed, which is considerably higher than the unemployment rate in the German population in general (8.6 % in 2010; source: German Federal Statistical Office). Different explanations of why obese individuals display a higher unemployment rate compared to non-obese counterparts are given in the literature. Firstly, obesity is a severely debilitating heath condition that negatively impacts productivity and employment and is a risk factor for a wide number of diseases [1, 50]. In our cohort, a high somatic symptom burden, depressive disorders and panic disorders were significantly more often among the unemployed. Additionally, obese individuals may have lower self-esteem and may experience discrimination at work [50, 51]. Furthermore, obesity may accelerate the departure from the labour market through early retirement [52]. In our study, 14.5 % of the participants were early retirees.

The PHQ was applied in a specially developed computer-based version. Completion of the electronic version took on average 8.5 minutes, which is somewhat shorter than completion time reported for the paper-pen version (approx. 10 minutes) [18]. Computerized tests are known to offer advantages over pencil-and-paper based assessment such as rapid completion time, high acceptance rates and immediate output of results [53–55]. The electronic documentation of the output files might mitigate or minimize diagnostic errors [56]. As skipping of a displayed question was impossible for the participant, all questions were answered and no values were missing. This is a relevant advantage of the computerized tests compared to paper-pen versions. Furthermore, computer-based tests facilitate repetitive monitoring and detection of change processes.

There are some limitations of the present study that need to be addressed. As the study population was recruited from patient information meetings, it is subject to selection bias and may therefore not be representative of bariatric surgery candidates on the whole. Approximately 63 % of the individuals who attended the information meeting about bariatric surgery decided to participate in the testing; the others did not participate predominantly because they were not interested in bariatric surgery. These individuals were not assessed surgically or for mental health disorders and we are left with speculation on the health of these individuals. As all patients who underwent bariatric surgery in the further course performed the PHQ testing, the study results provide a comprehensive picture of prebariatric patients.

Psychiatric syndromes obtained from the PHQ relied on patient self-report and participants might answer with the intention not to jeopardise bariatric surgery. Furthermore, the PHQ does not screen for personality disorders, which may contribute to the bariatric outcome (e.g. borderline disorders) [11, 57]. Finally, the PHQ is so far not validated in morbidly obese patients and somatic complaints might have biased reports on somatoform or affective disorders. To overcome these shortcomings, all patients underwent a semi-structured interview administered by a psychosomatic specialist (co-author VP) experienced in the evaluation of bariatric patients prior to surgery. If a participant was diagnosed positive for one or more syndromes, the psychosomatic specialist determined if the severity of a mental health disorder was a contraindication for bariatric surgery. Then the most appropriate therapy plan was discussed, which included nutrition counselling, outpatient or in-patient psychotherapy.

## Conclusions

Morbidly obese individuals seeking bariatric treatment are known to have high prevalence rates of mental health disorders. Our data suggest the PHQ be suited as a screening instrument for clinically relevant mental health disorders. Embedded in a computerized environment its application may help manage repeated and easy testing performed by non-mental health specialists. It may thus bridge existing diagnostic shortcomings in a bariatric clinical context. There is no doubt that results obtained with this test will need to be complemented by additional social and physical assessments to respect the broad scope of the complex health situation governing morbid obesity.

## Abbreviations

PHQ: Patient Health Questionnaire
BMI: Body mass index
